# Atherogenic Lipid Indices in Colorectal Cancer: Metabolic Associations and Survival Outcomes

**DOI:** 10.3390/diagnostics16050810

**Published:** 2026-03-09

**Authors:** Răzvan Alexandru Marinescu, Daniela Marinescu, Lidia Boldeanu, Ana-Maria Ciurea, Marius Bică, Ștefan Pătrașcu, Victor Dan Eugen Strâmbu, Petru Adrian Radu, Petrica Popa, Mohamed-Zakaria Assani, Mihail Virgil Boldeanu, Valeriu Șurlin

**Affiliations:** 1 Doctoral School, University of Medicine and Pharmacy of Craiova, 200349 Craiova, Romania; razvanalexandrumarinescu98@gmail.com (R.A.M.); mohamed.assani@umfcv.ro (M.-Z.A.); 2 Department of Surgery, University of Medicine and Pharmacy of Craiova, 200349 Craiova, Romania; daniela.marinescu@umfcv.ro (D.M.); marius.bica@umfcv.ro (M.B.); vsurlin@gmail.com (V.Ș.); 3 Department of Microbiology, Faculty of Medicine, University of Medicine and Pharmacy of Craiova, 200349 Craiova, Romania; 4 Department of Oncology, University of Medicine and Pharmacy of Craiova, 200349 Craiova, Romania; amciurea14@gmail.com; 5 Department of Surgery, University of Medicine and Pharmacy “Carol Davila”, 020021 Bucharest, Romania; victor.strambu@umfcd.ro (V.D.E.S.); petru.radu@umfcd.ro (P.A.R.); 6 Department of Gastroenterology, University of Medicine and Pharmacy of Craiova, 200349 Craiova, Romania; petrica.popa@umfcv.ro; 7 Department of Immunology, Faculty of Medicine, University of Medicine and Pharmacy of Craiova, 200349 Craiova, Romania; mihail.boldeanu@umfcv.ro

**Keywords:** colorectal cancer, type 2 diabetes mellitus, atherogenic indices, AIP, TyG, triglyceride-to-HDL ratio, remnant cholesterol, survival, prognosis

## Abstract

**Background/Objectives:** Type 2 diabetes mellitus (T2DM) and atherogenic dyslipidemia have been implicated in colorectal cancer (CRC) development, but their prognostic relevance after cancer diagnosis remains unclear. This study aimed to evaluate the association between T2DM, lipid-derived atherogenic indices, and survival outcomes in patients with CRC. **Methods:** We conducted a retrospective cohort study including 240 CRC patients, of whom 60 had coexisting T2DM. Overall survival (OS) and disease-free survival (DFS) were analyzed using the Kaplan–Meier (KM) method and log-rank tests. In the absence of recurrence-specific data, DFS was defined as time to death or last follow-up. Lipid-related indices, including the atherogenic index of plasma (AIP), atherogenic coefficient (AC), remnant cholesterol (RC), non-high-density lipoprotein cholesterol (non-HDL-C), triglyceride–glucose (TyG) index, and triglyceride-to-HDL cholesterol ratio (TG/HDL-C), were evaluated by tertiles in KM analyses. Multivariable Cox proportional hazards models were constructed to assess the independent prognostic value of AIP, AC, and RC (entered separately as a continuous variable standardized to 1 standard deviation), adjusted for age, sex, adjuvant chemotherapy, radiotherapy, and T2DM status. Sensitivity analyses were performed in stage III–IV patients. **Results:** During follow-up, 28 deaths occurred. OS did not differ significantly between CRC patients and those with CRC coexisting with T2DM (log-rank *p*-values = 0.220). DFS analyses showed no significant differences across tertiles of any lipid-related index (all log-rank *p*-values > 0.05), with overlapping survival curves and no consistent dose–response patterns. In adjusted Cox models, AIP (hazard ratio [HR] per 1 SD = 0.71, 95% CI 0.48–1.06), AC (HR = 0.72, 95% CI 0.44–1.20), and RC (HR = 0.66, 95% CI 0.39–1.12) were not independently associated with DFS. Results were consistent in advanced-stage disease (stage III–IV). **Conclusions:** In this cohort of patients with CRC, neither T2DM nor lipid-derived indices reflecting atherogenic dyslipidemia and insulin resistance were independently associated with OS or DFS. These findings help refine the clinical interpretation of lipid-derived biomarkers in CRC, suggesting limited prognostic utility beyond established oncologic factors.

## 1. Introduction

Colorectal cancer (CRC) remains one of the leading causes of cancer morbidity and mortality worldwide, representing the third most commonly diagnosed cancer and the second leading cause of cancer-related deaths globally [1]. Despite advances in screening, molecular stratification, and therapeutic approaches, prognosis continues to vary substantially across patients with apparently similar tumor stages, suggesting that additional biological factors may modulate disease behavior beyond conventional TNM classification [2]. Increasing evidence indicates that patient metabolic status, particularly obesity, insulin resistance (IR), chronic inflammation, and atherogenic dyslipidemia, plays a central role in CRC initiation, progression, and survival outcomes [3].

Type 2 diabetes mellitus (T2DM) is a well-established risk factor for CRC, with meta-analyses showing a 27–35% higher incidence and worse overall survival among individuals with diabetes compared to non-diabetic CRC patients [4]. Hyperinsulinemia, elevated insulin-like growth factor 1 signaling, pro-oxidative stress, and low-grade inflammation appear to promote carcinogenesis and metastatic potential synergistically [5]. Moreover, T2DM is frequently accompanied by atherogenic dyslipidemia (high triglycerides (TG), low high-density lipoprotein cholesterol (HDL-C), elevated low-density lipoprotein cholesterol (LDL-C) remnants), which has itself been implicated in tumor proliferation, altered membrane signaling, immune dysregulation, and impaired chemotherapy responsiveness [6].

In recent years, simple lipid-derived indices, such as the atherogenic index of plasma (AIP), atherogenic coefficient (AC), Castelli risk ratios, triglyceride-to-HDL-C ratio (TG/HDL-C), non-HDL-C, and the triglyceride–glucose index (TyG), have gained substantial attention as integrative markers reflecting IR, lipoprotein metabolism, and cardiometabolic risk [7,8,9,10,11,12,13,14]. In addition to these indices, the Visceral Adiposity Index (VAI) has emerged as a composite marker integrating anthropometric and metabolic parameters (waist circumference (WC), body mass index (BMI), TG, and HDL-C) to estimate visceral fat dysfunction. Recent evidence highlights its association with cardiometabolic risk and systemic metabolic alterations, further supporting the relevance of lipid-derived and adiposity-related indices in chronic disease research [15,16,17].

These indices may offer prognostic value in oncology, as dysregulated lipid metabolism is increasingly recognized as a hallmark of cancer, influencing tumor cell proliferation, membrane fluidity, mitochondrial energetics, and inflammatory signaling. Preliminary evidence indicates that elevated AIP, a high TG/HDL-C ratio, and an increased TyG index are associated with more advanced CRC stage, greater metastatic potential, and poorer survival outcomes [18,19]. However, these findings remain inconsistent across studies due to heterogeneous populations, limited follow-up duration, and small event counts, particularly in early-stage disease.

The interaction between CRC and T2DM may further amplify the prognostic implications of dyslipidemia. Patients with CRC coexisting with T2DM exhibit higher rates of postoperative complications, recurrence, and mortality compared with non-diabetic individuals, partly due to metabolic inflexibility, impaired immunity, endothelial dysfunction, and chronic pro-inflammatory signaling [20]. Despite this, few studies have evaluated whether lipid-derived atherogenic indices, such as TyG, differ meaningfully between CRC patients and those with CRC coexisting with T2DM [21]. No standardized framework currently exists to integrate such metabolic biomarkers into CRC risk stratification.

Although numerous studies have investigated the impact of T2DM on CRC risk and prognosis, very few have examined the metabolic and lipidomic differences between CRC patients and those with CRC coexisting with T2DM. The existing literature predominantly focuses on conventional lipid parameters (LDL-C, HDL-C, TG). At the same time, advanced atherogenic indices such as AIP, TyG, TG/HDL, and RC remain largely unexplored in this clinical context.

To our knowledge, no study has systematically compared derived lipid indices between CRC patients and those with CRC coexisting with T2DM, nor has it evaluated their association with clinical stage, tumor characteristics, or disease-free survival (DFS). This represents a significant knowledge gap, as these indices may better reflect IR, systemic inflammation, and metabolic reprogramming—mechanisms that are increasingly recognized as contributors to cancer progression.

The purpose of this study was to evaluate the association between T2DM and lipid-derived atherogenic indices (AIP, AC, remnant cholesterol (RC), TG/HDL-C, non-HDL-C, and TyG index) with overall survival (OS) and DFS in CRC patients and those with CRC coexisting with T2DM. Specifically, we aimed to determine whether composite lipid-related markers reflecting atherogenic dyslipidemia and IR provide independent prognostic information beyond established clinical and treatment-related factors.

## 2. Materials and Methods

### 2.1. Patient Selection

We conducted a retrospective cohort study between January 2023 and October 2025 at a single university hospital in Craiova, Dolj, Romania, including 240 patients with CRC, of whom 60 had T2DM and 180 did not. The study complied with the Declaration of Helsinki and was approved by the Ethics Committee of the Emergency County Clinical Hospital of Craiova and the Institutional Ethics Committee of the University of Medicine and Pharmacy of Craiova, Romania (no. 235/4 October 2023).

Patients were eligible for inclusion in the study if they met all of the following criteria:

• Histologically confirmed CRC, irrespective of tumor location (colon or rectum) according to the latest criteria developed by the World Health Organization (WHO) working group for tumors of the digestive system [22];
• Age ≥ 18 years at the time of CRC diagnosis;
• Availability of baseline clinical and laboratory data at diagnosis or prior to initiation of oncologic treatment, including lipid profile parameters required for the calculation of lipid-derived indices (AIP, AC, RC, non-HDL-C, TyG, and TG/HDL-C);
• Known T2DM status, defined according to documented medical history or antidiabetic treatment at the time of CRC diagnosis;
• Documented follow-up data allowing assessment of survival outcomes, including date of diagnosis, date of death, or last known follow-up;
• Patients treated according to standard oncologic protocols, including surgery with or without adjuvant chemotherapy and/or radiotherapy, as clinically indicated.

Patients were excluded from the study if any of the following criteria were present:

• Type 1 diabetes mellitus or other specific forms of diabetes, including secondary diabetes due to pancreatic disease or medication-induced diabetes.
• History of another malignant disease diagnosed within the previous five years, except for adequately treated non-melanoma skin cancer.
• Severe acute inflammatory or infectious conditions at the time of lipid assessment that could significantly alter lipid metabolism.
• Chronic liver disease (e.g., cirrhosis, active hepatitis) or end-stage renal disease, which may substantially affect lipid and glucose metabolism.
• Use of lipid-lowering therapy initiated after CRC diagnosis but before lipid assessment if baseline lipid values were not available.
• Incomplete or missing key data, including unavailable lipid parameters, unknown T2DM status, or lack of follow-up information.
• Patients lost to follow-up immediately after diagnosis, precluding meaningful survival analysis.

### 2.2. Evaluation of Diabetes

Diabetes is diagnosed if any of the following are true: a medical diagnosis by healthcare providers, glycated hemoglobin A1c (HbA1c) above 6.5%, fasting plasma glucose (FPG) of 7.0 mmol/L or higher, random blood glucose of 11.1 mmol/L or higher, or a two-hour blood glucose over 11.1 mmol/L after an oral glucose tolerance test (OGTT). It may also be diagnosed if a random glucose test shows hyperglycemia and hyperglycemic symptoms such as polyuria, polydipsia, weight loss, or hyperglycemic crises are present [23].

### 2.3. Assessment of Biometric Parameters

We calculated the BMI from the participants’ height and weight. The formula used is BMI = weight (kilograms)/height^2^ (meters), according to the WHO criteria [24]. We measured weight with a scale and height using a measuring stick attached to the scale.

Hip circumference (HC) was measured around the femoral trochanters, while WC was taken at the midpoint between the upper iliac crest and the lower rib cage.

### 2.4. Evaluation of Various Indices

To evaluate IR and the metabolic profile, we used the formulas in Table 1.

### 2.5. Laboratory Investigations

After acquiring the anthropometric data, we conducted more thorough evaluations of the subjects in the lab.

#### 2.5.1. Sample Collection

Each patient provided two additive-free tubes containing ~5 mL of venous blood (Becton, Dickinson and Company, Franklin Lakes, NJ, USA). After collection, samples were processed per standard procedures: centrifuged in a Hermle centrifuge (Hermle AG, Gosheim, Baden-Württemberg, Germany) at 3000× *g* for 10 min within 4 h of collection and after clotting. Serum from one tube was aliquoted into pre-labeled vials, sealed to prevent contamination, and stored at −20 °C to −80 °C. Freeze–thaw cycles were avoided. Frozen serum was thawed at room temperature before analysis. Immunological tests used these aliquots, while biochemical analyses used serum from the second tube.

#### 2.5.2. Biochemical Investigations

Biochemical parameters were measured using the ARCHITECT c4000 clinical chemistry analyzer (Abbott Laboratories, Abbott Park, IL, USA), which utilizes photometric, enzymatic, and potentiometric techniques depending on the analyte. Serum or plasma samples were analyzed according to the manufacturer’s standard operating procedures. All measurements adhered to the calibration and quality control protocols specified for the equipment.

### 2.6. Statistical Analysis

Statistical analyses were performed using GraphPad Prism 11.0.0 (84) (GraphPad Software, San Diego, CA, USA) and Microsoft Excel for data preprocessing. Continuous variables were inspected for missingness, distributional properties, and outlier influence before inferential analyses. Normality was evaluated using the Shapiro–Wilk test and assessment of histograms and Q–Q plots. Depending on distribution, continuous variables were expressed as mean ± SD or median [IQR], whereas categorical variables were described as frequencies and percentages.

Between-group comparisons were conducted among patients with CRC, stratified by T2DM status. Differences in continuous variables were assessed using the independent samples t-test for normally distributed data or the Mann–Whitney U test for non-normally distributed data. Categorical variables were compared using the chi-square test or Fisher’s exact test where appropriate. Lipid-derived indices (AIP, AC, RC, TG/HDL-C, non-HDL-C, and TyG) were calculated directly from laboratory values using standard formulas.

DFS was defined as the time from CRC diagnosis to death from any cause or last follow-up and was expressed in months. Survival time was derived from the automatically calculated variable (OS_months), and event status was defined as death (status = 1) or censoring at last follow-up (status = 0). In the absence of recurrence-specific data, this operational definition of DFS partially overlaps with OS, potentially limiting the ability to detect associations specific to tumor recurrence.

Patients were stratified according to their coexisting T2DM status. Kaplan–Meier survival analyses were performed to evaluate OS by T2DM status and DFS by lipid-related indices. For DFS analyses, lipid-derived indices, including AIP, AC, RC, non-HDL-C, TyG, and TG/HDL-C, were categorized into tertiles based on group-specific distributions (CRC vs. CRC coexisting with T2DM). Differences in DFS across tertiles were assessed using the log-rank test.

To evaluate the independent prognostic value of lipid-derived indices, Cox proportional hazards regression models were constructed. To avoid multicollinearity, AIP, AC, and RC were entered separately, one index per model, as continuous variables standardized per 1-SD increase. Multivariable models were adjusted for age, sex, adjuvant chemotherapy, radiotherapy, and T2DM status. Hazard ratios (HRs) and 95% confidence intervals (CIs) were reported. Given that all DFS events occurred in patients with stage III–IV diseases, sensitivity analyses were additionally performed by restricting Cox models to patients with advanced-stage colorectal cancer. Statistical significance was defined as a two-sided *p*-value < 0.05.

## 3. Results

### 3.1. General Characteristics of Patients

The cohort included 240 patients with CRC, of whom 60 (25.00%) had coexisting T2DM. Table 2 presents the baseline demographic, clinical, and tumor-related characteristics of patients with CRC, stratified by T2DM status (CRC vs. CRC coexisting with T2DM). Overall, the two groups were comparable in several demographic domains, but meaningful metabolic and tumor-related differences were observed.

Age and sex distribution did not differ significantly between groups (*p* > 0.05), indicating that the cohorts were demographically balanced and that potential metabolic differences are unlikely to be confounded by age structure or sex ratio.

Lifestyle factors, including smoking and alcohol consumption, were also similar between the two groups (*p* > 0.05), suggesting that these exposures do not explain downstream biochemical differences.

In contrast, hypertension was significantly more prevalent in the CRC coexisting with T2DM group compared with non-diabetic patients, consistent with the clustering of cardiometabolic comorbidities typically seen in individuals with long-standing diabetes.

Regarding tumor characteristics, significant differences emerged in local tumor invasion. Both groups showed a markedly higher proportion of T3–T4 (more than 82%).

Nodal involvement (N stage) did not differ substantially between groups, indicating that the extent of lymphatic spread was comparable.

Interestingly, distant metastases (M stage) were more frequently observed in the non-diabetic group (M1 cases) compared with the T2DM group. This pattern may reflect differences in stage distribution, referral patterns, or timing of diagnosis; it does not necessarily indicate a protective effect of diabetes.

The distribution of overall TNM stages also differed: CRC patients showed a higher frequency of stage II and IV diseases, whereas CRC coexisting with T2DM patients had proportionally more stage III tumors. Although these patterns are descriptive relevant, inferential interpretation should remain cautious due to unequal subgroup sizes.

With respect to tumor grade, poorly differentiated tumors (G3) were more common in the CRC coexisting with T2DM group, suggesting a potential link between diabetes-related metabolic dysregulation and more aggressive histology.

Tumor sidedness did not differ significantly (*p* > 0.05), with both cohorts showing a predominance of left-sided tumors.

Finally, oncologic treatments (chemotherapy, radiotherapy) were similarly distributed between groups, confirming comparable therapeutic exposure, while—as expected—patients with T2DM demonstrated complete or near-complete use of antidiabetic therapies (metformin, insulin, sodium-glucose cotransporter-2 inhibitors (SGLT2i), glucagon-like peptide-1 receptor agonists (GLP-1RAs)).

### 3.2. Baseline Metabolic Characteristics According to T2DM Status (CRC vs. CRC Coexisting with T2DM)

Table 3 compares metabolic and lipid-related biomarkers between CRC patients and those with CRC coexisting with T2DM. Statistically significant differences were observed across nearly all evaluated parameters, indicating two distinct metabolic phenotypes.

Although both groups fall within the overweight range, CRC patients had a significantly higher BMI (*p* = 0.002). This suggests that general adiposity was not the primary driver of metabolic derangements in the diabetic group, which was more strongly influenced by glycemic and lipid abnormalities. Despite lower BMI, CRC coexisting with T2DM patients exhibited significantly higher WC, indicating greater central adiposity, a hallmark of IR and cardiometabolic risk. This pattern aligns with the metabolic syndrome phenotype commonly observed in T2DM.

As expected, HbA1c levels were markedly higher in the CRC coexisting with T2DM group, confirming poor long-term glycemic control and supporting the classification of this cohort as metabolically high risk. Fasting glucose levels also differed significantly, highlighting active hyperglycemia in the CRC coexisting with T2DM group, consistent with their markedly elevated HbA1c.

Total cholesterol (TC) was significantly lower in the diabetic group, likely reflecting the combined effects of statin therapy, altered hepatic lipid metabolism, and diabetes-related dyslipidemia. LDL-C was also lower in diabetic patients, possibly reflecting higher statin use and the shift from LDL particles toward triglyceride-rich lipoproteins, as seen in IR. HDL-C was significantly reduced in the CRC coexisting with T2DM group. The combination of lower HDL-C and higher triglycerides corresponds to the classic atherogenic dyslipidemia pattern associated with T2DM. Non-HDL-C, a superior predictor of cardiovascular risk compared with LDL-C alone, was markedly lower in the diabetic group. This parallels the reductions seen in TC and LDL-C, again likely influenced by statin therapy and altered lipid metabolism. Triglyceride levels were significantly higher in diabetic patients, reinforcing the IR, hypertriglyceridemic phenotype, and setting the stage for elevated derived indices such as AIP, TG/HDL-C, RC, and TyG.

### 3.3. Lipid-Derived Indices in CRC and Those with CRC Coexisting with T2DM Patients

Table 4 compares six atherogenic and insulin-resistance-related indices between the CRC and the CRC coexisting with T2DM groups. Significant metabolic differences were observed in most indices, indicating two distinct biochemical phenotypes.

AIP was significantly higher in the T2DM group, reflecting the combination of elevated TG and reduced HDL-C typically seen in IR. This aligns closely with their higher TG/HDL ratio and TyG index. AC did not differ significantly between groups.

RC levels were significantly lower in the T2DM group, which may seem counterintuitive given their higher TG. This pattern likely reflects lower LDL-C and TC from widespread statin therapy in the CRC coexisting with T2DM group, and greater variability in remnant-rich lipoproteins, as indicated by the wider IQR. This suggests that RC may not track perfectly with TG in CRC coexisting with T2DM, and that its prognostic relevance may differ by metabolic phenotype.

TG/HDL-C ratio was significantly higher, and non-HDL-C was markedly lower in the CRC coexisting with T2DM group, driven primarily by lower LDL-C and TC. This pattern again highlights that diabetic dyslipidemia is dominated by high TG and low HDL-C, rather than elevated LDL-C, leading to discrepancies across indices that depend on cholesterol fractions. Also, TyG was significantly higher in patients with CRC coexisting with T2DM, reflecting severe IR.

### 3.4. Comparing Lipid-Derived Indices According to Gender in CRC and Those with CRC Coexisting with T2DM Patients

Table 5 assesses whether gender affects atherogenic and IR-related indices in CRC patients, analyzed separately for the CRC and the CRC coexisting with T2DM groups.

Across all six lipid-derived indices and in both metabolic subgroups (CRC and CRC coexisting with T2DM), gender did not influence the atherogenic or IR profile. This finding suggests that sex does not act as a confounder for lipid-derived indices in this CRC cohort, metabolic dysregulation appears primarily driven by diabetes status, not by gender, and both males and females exhibit similar lipid remodeling and IR patterns within each disease subgroup.

### 3.5. Lipid Profile According to Tumor Stage

Table 6 evaluates lipid-derived indices across TNM stages (I–IV) in the CRC and the CRC coexisting with T2DM groups using a two-way ANOVA framework, where

• Row factor reflects between-subject variability unrelated to TNM stage (individual-level heterogeneity).
• Column factor reflects the effect of TNM stage on lipid-derived indices.

In the *CRC group*, tumor stage selectively influences triglyceride-driven and IR-related indices, but not cholesterol-based markers.

• Row Factor

For all lipid-derived indices (AIP, AC, RC, TG/HDL, non-HDL-C, TyG), the row factor was not statistically significant (all *p* > 0.25). This indicates that inter-individual variability did not significantly influence the distribution of lipid indices beyond what is explained by TNM stage grouping.

In practical terms, this suggests a relatively homogeneous metabolic background within the non-diabetic CRC cohort once stratified by cancer stage.

• Column Factor (Effect of TNM Stage)

AIP showed a statistically significant column effect (F(3,102) = 4.122, *p* = 0.008), indicating that AIP varies across TNM stages. AIP values increased from stage I to stages II–III and decreased again in stage IV, suggesting a non-linear relationship between atherogenic dyslipidemia and tumor progression.

RC also demonstrated a significant column effect (F(3,102) = 2.889, *p* = 0.039). RC peaked in stages II–III and declined in stage IV, suggesting metabolic remodeling during intermediate disease stages, followed by lipid depletion or treatment effects in advanced disease. AC, TG/HDL, and non-HDL-C did not show statistically significant column effects (*p* = 0.064, 0.183, and 0.362, respectively), suggesting that cholesterol-dependent indices are less sensitive to tumor stage progression in CRC patients.

The TyG index showed a borderline significant column effect (F(3,102) = 2.712, *p* = 0.049), indicating increasing IR from early to intermediate stages, with partial attenuation in stage IV.

In the *CRC coexisting with T2DM group*, lipid-derived indices are dominated by diabetes-related metabolic heterogeneity, and TNM stage has no independent effect:

• Row Factor

In contrast to the CRC group, the row factor was statistically significant for AC (F(26,30) = 2.120, *p* = 0.025), indicating substantial inter-individual metabolic variability in AC values among diabetic patients, independent of TNM stage.

For all other indices (AIP, RC, TG/HDL, non-HDL-C, TyG), the row factor was not significant, suggesting moderate within-group heterogeneity.

This pattern reflects the metabolic complexity of T2DM, in which lipid indices—particularly AC—are strongly influenced by individual factors, such as diabetes duration, treatment, and glycemic control.

• Column Factor (Effect of TNM Stage)

None of the lipid-derived indices in the CRC coexisting with the T2DM group showed a significant column effect (all *p* > 0.21). This indicates that TNM stage does not significantly alter lipid-derived indices in CRC coexisting with T2DM patients. Even though median or mean values tended to increase from stages I to III for indices such as AIP, TG/HDL, and TyG, these trends did not reach statistical significance, likely due to a smaller sample size, wide inter-individual variability, and strong confounding by diabetes-related metabolic factors. These findings suggest that in CRC patients, TNM stage significantly influenced AIP, RC, and TyG index values. In contrast, in CRC coexisting with T2DM patients, no lipid-derived index varied significantly across tumor stages, indicating that diabetes-related metabolic heterogeneity predominates over stage-dependent effects.

### 3.6. Survival Analysis Results (DFS and OS)

During the follow-up period, a total of 28 deaths were recorded in the entire cohort. Overall survival (OS) and disease-free survival (DFS), defined in the present analysis as time to death or last follow-up, were evaluated according to T2DM status and lipid-related indices.

#### 3.6.1. OS According to T2DM Status

**Figure 1:**
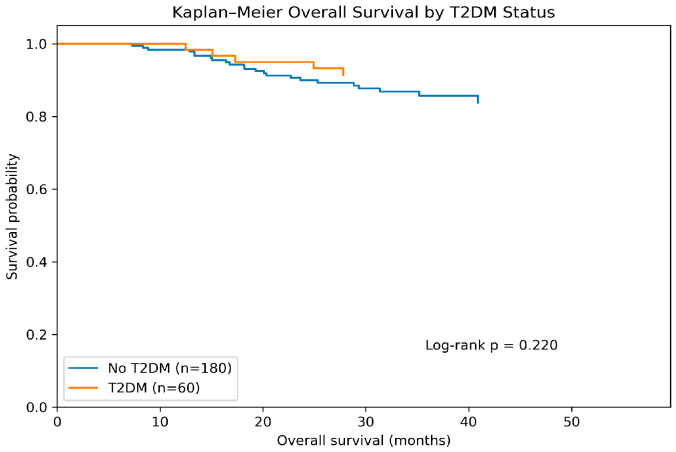
Kaplan–Meier curves illustrating overall survival (OS) in patients with colorectal cancer (CRC) stratified by type 2 diabetes mellitus (T2DM) status. Patients without T2DM (*n* = 180, 23 events) and those with T2DM (*n* = 60, 5 events) were compared using the log-rank test. No statistically significant difference in survival was observed between the two groups (log-rank *p* = 0.220).

Kaplan–Meier analysis demonstrated no statistically significant difference in OS between the CRC and the CRC coexisting with T2DM groups (log-rank test χ^2^ = 1.504, *p* = 0.220) (Figure 1). Median OS was not reached in either subgroup (Table 7). Although survival probabilities are presented at predefined follow-up time points (12, 24, 36, 48, and 60 months) for CRC patients and CRC coexisting with T2DM, they tended to be numerically higher in CRC coexisting with T2DM patients at later time points; however, this difference did not reach statistical significance.

Although survival probabilities appeared numerically higher in the CRC coexisting with T2DM group at later time points, the wide overlap of confidence intervals and the limited number of events precluded statistical significance.

#### 3.6.2. Disease-Free Survival According to Lipid-Related Indices

**Figure 2:**
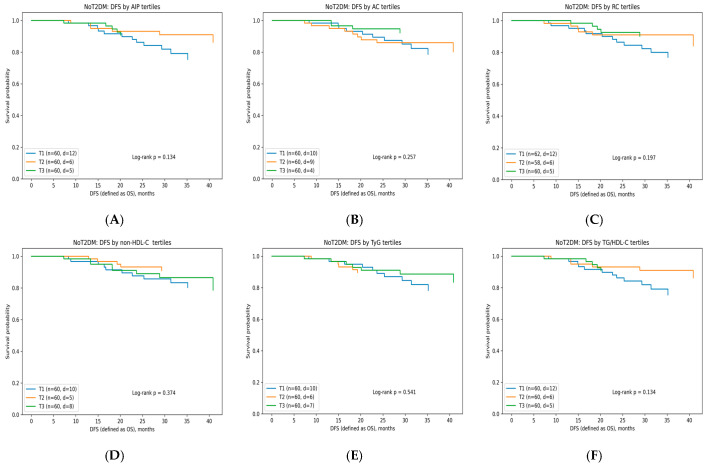
Kaplan–Meier DFS according to tertiles of lipid-derived indices in CRC patients: (**A**) atherogenic index of plasma (AIP) (log10[TG/HDL]); (**B**) atherogenic coefficient ((TC–HDL)/HDL); (**C**) remnant cholesterol (mg/dL); (**D**) non-HDL cholesterol (mg/dL); (**E**) triglyceride–glucose index (TyG) (ln[TG·Glucose/2]); and (**F**) triglyceride-to-HDL-C ratio (TG/HDL-C). Curves show substantial overlap, indicating no significant differences between groups.

**Figure 3:**
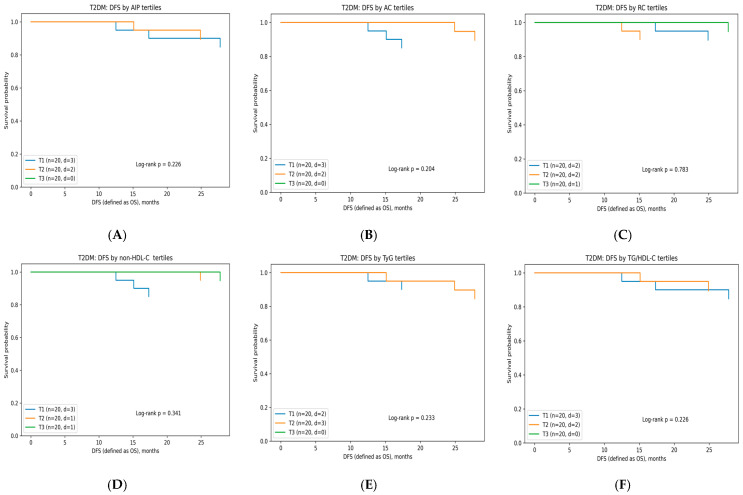
Kaplan–Meier DFS according to tertiles of lipid-derived indices in CRC coexisting with T2DM patients: (**A**) atherogenic index of plasma (AIP) (log10[TG/HDL]); (**B**) atherogenic coefficient ((TC–HDL)/HDL); (**C**) remnant cholesterol (mg/dL); (**D**) non-HDL cholesterol (mg/dL); (**E**) triglyceride–glucose index (TyG) (ln[TG·Glucose/2]); and (**F**) triglyceride-to-HDL-C ratio (TG/HDL-C). Curves show substantial overlap, indicating no significant differences between groups.

DFS analyses were performed separately in CRC patients (*n* = 180, 23 events) and in those with CRC coexisting with T2DM (*n* = 60, 5 events) (Table 8). Kaplan–Meier analyses demonstrated no statistically significant differences in DFS across tertiles of AIP, AC, RC, non-HDL-C, TyG, or TG/HDL-C in either metabolic subgroup (all log-rank *p* > 0.05) (Figure 2A–F and Figure 3A–F). Importantly, no consistent dose–response pattern across tertiles was observed for any lipid-derived index in either metabolic subgroup, further supporting the absence of a clinically meaningful association with DFS.

To minimize multicollinearity and overfitting in the context of a limited number of events, lipid-derived indices were evaluated in separate Cox models and standardized per 1-SD increase:

• Model A (AIP): DFS ~ AIP + Age + Sex + Chemo + Radiotherapy + T2DM
• Model B (AC): DFS ~ AC + Age + Sex + Chemo + Radiotherapy + T2DM
• Model C (RC): DFS ~ RC + Age + Sex + Chemo + Radiotherapy + T2DM

#### 3.6.3. Cox Proportional Hazards Regression Analyses

In multivariable Cox proportional hazards models including all patients (*n* = 240), none of the lipid-derived indices, evaluated as continuous variables, per 1-SD increase, were independently associated with DFS.

Specifically, AIP showed a non-significant trend toward a lower risk of DFS events (HR per 1 SD = 0.714, 95% CI: 0.480–1.064, *p* = 0.098), while AC (HR = 0.722, 95% CI: 0.436–1.196, *p* = 0.206) and RC (HR = 0.660, 95% CI: 0.389–1.120, *p* = 0.123) were not significantly associated with DFS.

Sensitivity analyses restricted to patients with stage III–IV CRC yielded consistent results. In these models, AIP (HR per 1 SD = 0.823, 95% CI: 0.548–1.236, *p* = 0.348), AC (HR = 0.848, 95% CI: 0.508–1.414, *p* = 0.527), and RC (HR = 0.780, 95% CI: 0.463–1.314, *p* = 0.350) were not independently associated with DFS (Table 9).

Overall, Cox regression analyses confirmed the findings of the Kaplan–Meier analyses, indicating that lipid-derived indices reflecting atherogenic dyslipidemia were not significant predictors of DFS in this cohort after adjustment for relevant clinical covariates.

#### 3.6.4. Multivariable Cox Proportional Hazards Models for DFS, Including Lipid-Derived Indices and Clinical Covariates

In the overall cohort (all patients; *n* = 240, *events* = 28), multivariable Cox proportional hazards models were used to evaluate whether lipid-derived indices were independently associated with DFS, adjusting for age, sex, adjuvant chemotherapy, radiotherapy, and T2DM status (Table 10). To avoid multicollinearity, AIP, AC, and RC were entered separately, each standardized by 1 SD.

Across these models, none of the lipid-derived indices showed a statistically significant association with DFS. AIP displayed a non-significant trend toward a lower hazard of DFS events (HR per 1 SD = 0.71, 95% CI 0.48–1.06, *p* = 0.098), while AC (HR = 0.72, 95% CI 0.44–1.20, *p* = 0.206) and RC (HR = 0.66, 95% CI 0.39–1.12, *p* = 0.123) were not significant predictors.

Age and male sex were not significantly associated with DFS in the overall cohort. In contrast, adjuvant chemotherapy was strongly associated with a higher hazard of DFS events (HR > 40, *p* < 0.001), most likely reflecting confounding by indication (i.e., chemotherapy being preferentially administered to patients with more advanced or aggressive disease). Radiotherapy was associated with a significantly lower hazard of DFS events (HR ≈ 0.22–0.25, *p* < 0.05), consistent with a protective effect in appropriately selected patients.

T2DM status was associated with a significantly reduced hazard of DFS events (HR = 0.21, 95% CI 0.06–0.73, *p* = 0.014) in models that included it. This apparent protective association should be interpreted cautiously, as it may reflect residual confounding and differences in surveillance or treatment patterns rather than a true biological advantage.

In patients with advanced CRC (stage III–IV; *n* = 120, *events* = 28), multivariable Cox proportional hazards models were constructed to evaluate the independent association between lipid-derived indices and DFS, adjusting for age, sex, adjuvant chemotherapy, radiotherapy, and T2DM status (Table 11). As in the overall cohort, AIP, AC, and RC were entered into the models separately as continuous variables standardized by 1 SD.

Across all models, none of the lipid-derived indices were significantly associated with DFS in patients with stage III–IV diseases. Specifically, AIP was not associated with DFS (HR per 1 SD = 0.82, 95% CI 0.55–1.24, *p* = 0.348), nor were AC (HR = 0.85, 95% CI 0.51–1.41, *p* = 0.527) or RC (HR = 0.78, 95% CI 0.46–1.31, *p* = 0.350). These findings indicate that lipid-derived indices do not exert a prognostic effect on DFS even when the analysis is restricted to patients with advanced-stage disease, in whom all DFS events occurred.

Age and male sex were not significantly associated with DFS in this subgroup. Adjuvant chemotherapy was strongly associated with an increased hazard of DFS events (HR = 21.80, 95% CI 3.86–123.14, *p* < 0.001), reflecting confounding by indication, as chemotherapy is preferentially administered to patients with more advanced or aggressive disease. Radiotherapy showed a non-significant trend toward a reduced hazard of DFS events (HR = 0.45, 95% CI 0.15–1.31, *p* = 0.143).

T2DM status remained significantly associated with a lower hazard of DFS events in advanced-stage patients (HR = 0.21, 95% CI 0.07–0.63, *p* = 0.006). This apparent protective association should be interpreted cautiously and is likely to reflect residual confounding, differences in clinical surveillance, or treatment-related factors rather than a true biological survival advantage conferred by diabetes.

Restricting the analysis to stage III–IV diseases did not reveal a prognostic role for lipid-derived indices, confirming that their lack of association with DFS is not driven by stage heterogeneity.

## 4. Discussion

In this retrospective observational study, we comprehensively evaluated conventional lipid parameters and lipid-derived indices reflecting atherogenic dyslipidemia and IR in patients with CRC, stratified by T2DM status. The main findings were (i) CRC coexisting with T2DM patients exhibited a more atherogenic and IR-related metabolic profile compared with CRC patients; (ii) triglyceride-driven indices (AIP, TG/HDL-C, and TyG) differed significantly according to diabetes status, and were more discriminatory than cholesterol-based indices; (iii) tumor stage was associated with variations in AIP, RC, and TyG index in CRC patients, but not in CRC coexisting with T2DM patients; and (iv) by integrating KM survival analyses with multivariable Cox proportional hazards models, neither T2DM status nor lipid-derived indices (AIP, AC, RC, non-HDL-C, TyG, and TG/HDL-C ratio) were independently associated with OS or DFS.

T2DM has been consistently associated with increased CRC risk and poorer oncologic outcomes in several meta-analyses and large observational studies. Pooled data indicate that patients with CRC coexisting with T2DM have higher all-cause and cancer-specific mortality compared with non-diabetic individuals. However, these studies showed substantial heterogeneity and often lack detailed adjustment for treatment, metabolic control, and comorbidity burden. Moreover, recent data suggest that diabetes severity and duration, rather than diabetes status alone, are key determinants of prognosis [20,29,30,30,31,32,33,34]. The absence of stratification by glycemic control or diabetes duration in our cohort may therefore have attenuated potential survival differences, highlighting the limitations of dichotomizing diabetes in survival analyses [35,36].

These findings provide important insights into the complex interplay among tumor biology, metabolic background, and lipid metabolism in CRC. Our results confirm that CRC coexisting with T2DM patients represents a metabolically distinct subgroup characterized by higher BMI, central adiposity, hyperglycemia, hypertriglyceridemia, and lower HDL-C levels. This phenotype is consistent with the well-established metabolic disturbances of T2DM and aligns with previous studies linking IR, hyperinsulinemia, and dyslipidemia to colorectal carcinogenesis and tumor progression [5,37,38]. Although TC and LDL-C levels were not markedly elevated in CRC coexisting with T2DM patients, likely influenced by lipid-lowering therapy, AIP, TG/HDL-C ratio, and TyG index were significantly higher. Triglyceride-driven indices were particularly sensitive to these differences, supporting their role as integrated markers of IR and metabolic dysregulation [7,8,9,10,11,12,13,14,39].

Large prospective cohort studies have shown that adverse lipid profiles are associated with increased CRC incidence. Elevated TG and reduced HDL-C levels have been linked to CRC incidence, supporting a role of dyslipidemia in colorectal carcinogenesis. However, these associations primarily relate to carcinogenesis rather than survival after diagnosis. Once malignancy is established, tumor stage and treatment appear to outweigh baseline metabolic characteristics in determining prognosis [40].

A novel finding of this study is tumor stage-dependent variation in lipid-derived indices, such as AIP, RC, and TyG index, among CRC patients, with higher values in intermediate stages (II–III). This pattern suggests dynamic metabolic remodeling during tumor progression. Triglyceride-rich lipoproteins and glucose metabolites support rapid tumor growth, explaining AIP and TyG’s sensitivity to tumor stage. The attenuation of these indices in stage IV may indicate cancer cachexia, systemic illness, or treatment-related metabolic changes. Cholesterol-based indices, such as AC and non-HDL-C, did not vary significantly across stages, indicating limited sensitivity to tumor metabolic changes. This finding aligns with studies showing pharmacologic interventions affect cholesterol but may not accurately reflect tumor-driven metabolic demands [41,42,43,44,45].

The absence of tumor stage-related variation in lipid-derived indices in CRC coexisting with T2DM patients suggests that chronic metabolic dysregulation and therapy-related effects may override tumor-specific metabolic signals. Persistent hyperinsulinemia, altered hepatic lipid metabolism, endothelial dysfunction, and chronic low-grade inflammation characterize T2DM and contribute to a relatively stable yet heterogeneous metabolic environment [5,31,37,38]. Instead, substantial inter-individual variability was observed, particularly for the AC. Some studies have reported that composite lipid ratios modestly improve survival prediction in resectable CRC, and that associations between lipid parameters and survival differ by tumor localization. These findings are inconsistent, often limited by reliance on traditional lipid fractions, lack of IR indices, and inadequate adjustment for metabolic conditions such as T2DM. These methodological differences may partly explain discrepancies with our results [46,47,48].

Despite these metabolic differences, survival analyses showed that lipid-derived indices lacked independent prognostic value (KM analysis of OS by T2DM status, log-rank *p* = 0.220). The KM curves overlapped across tertiles in the CRC and the CRC coexisting with T2DM groups, with no consistent trend suggesting better or worse survival with higher atherogenic index values. This pattern was confirmed in multivariable Cox models in which AIP, AC, and RC were entered individually as continuous variables, standardized to 1 SD. None of these indices were significantly associated with DFS after adjustment for age, sex, adjuvant chemotherapy, radiotherapy, and T2DM status, and trends observed in the overall analysis were attenuated in sensitivity analyses restricted to advanced (stage III–IV) disease. Notably, treatment-related variables exerted a substantially stronger influence on DFS than metabolic markers in all patients. Adjuvant chemotherapy was associated with markedly increased hazard ratios, reflecting confounding by indication, as patients receiving chemotherapy typically have more advanced or biologically aggressive disease. Conversely, radiotherapy was consistently associated with a reduced hazard of DFS events, supporting its protective role in appropriately selected patients. These findings underscore that, in the overall CRC population, therapeutic and disease-related factors, rather than baseline lipid-related metabolic characteristics, determine survival. Also, in advanced-stage patients, adjuvant chemotherapy remained strongly associated with DFS events, again reflecting confounding by indication, while radiotherapy showed a non-significant trend toward improved DFS. Importantly, the persistence of null associations for AIP, AC, and RC in this high-risk subgroup reinforces the conclusion that lipid-derived indices provide no incremental prognostic information beyond established clinical and treatment-related factors, even in patients with biologically aggressive disease. Taken together, the findings from multivariable Cox models consistently demonstrate that lipid-derived indices reflecting atherogenic dyslipidemia and IR do not independently influence DFS in CRC. The concordance between analyses performed in the overall cohort and those restricted to stage III–IV diseases strengthens the robustness of these results and argues against a clinically meaningful prognostic role for these metabolic markers.

Biologically, this is plausible. Lipid-derived indices primarily reflect systemic cardiometabolic status rather than intrinsic tumor aggressiveness or treatment responsiveness. While metabolic dysfunction may contribute to CRC initiation, its influence on survival appears limited once cancer is diagnosed and treated [33,40,49,50,51,52]. Moreover, systemic lipid levels and composite indices such as AIP often reflect cardiovascular risk rather than tumor-specific biology; indeed, the prognostic utility of these indices in cancer remains underexplored and inconsistent in the literature.

The apparent protective association of T2DM with survival observed in the adjusted Cox models is counterintuitive and should be interpreted with considerable caution. Given the limited number of events, particularly in the CRC coexisting with T2DM group, the estimate may be unstable and susceptible to random variation. In addition, residual confounding, collider bias, and differences in clinical surveillance or treatment allocation may have influenced this association. Therefore, this finding should not be interpreted as evidence of a biological survival advantage conferred by diabetes but rather as a potential statistical artifact requiring confirmation in larger, adequately powered cohorts.

From a clinical perspective, our findings suggest that lipid-derived indices, such as AIP, AC, RC, non-HDL-C, TyG, and TG/HDL-C, should not be considered prognostic biomarkers for survival in CRC patients. Although these markers remain valuable for cardiometabolic risk stratification and overall health management, they appear to have limited relevance for oncologic prognostication. Clinical decision-making should therefore continue to rely primarily on tumor-related factors and treatment response, while metabolic abnormalities should be addressed mainly in the context of cardiovascular prevention and long-term patient management [53,54,55].

### 4.1. Strengths and Limitations

Several limitations should be acknowledged when interpreting these findings. First, the relatively small number of survival events (28 overall, including only 5 in the CRC coexisting with T2DM group) limited statistical power and reduced the precision of HR estimates, particularly in multivariable models. To mitigate overfitting, lipid-derived indices were entered separately into Cox regression analyses rather than simultaneously. Nevertheless, the limited number of events may have constrained the ability to detect modest associations.

Second, because recurrence-specific data were unavailable, DFS was operationally defined as equivalent to OS, potentially underestimating associations specific to cancer recurrence.

Third, lipid-derived indices were assessed only at baseline, precluding evaluation of longitudinal metabolic changes. In addition, the lack of detailed information regarding diabetes duration, glycemic control, and diabetes-related complications may have attenuated potential associations between metabolic dysfunction and survival, as prior evidence suggests that these factors are more prognostically relevant than diabetes status alone. Moreover, detailed and standardized data on statin therapy, specific antidiabetic medications (including metformin, GLP-1 receptor agonists, and SGLT2 inhibitors), and longitudinal HbA1c levels were not systematically available in this retrospective oncologic cohort. These factors may influence both lipid metabolism and cancer outcomes, potentially introducing residual confounding.

Finally, residual confounding related to antidiabetic and lipid-lowering therapies cannot be excluded. Future studies incorporating longitudinal metabolic assessments and recurrence-specific endpoints are warranted to further elucidate the complex interplay between metabolic disease and CRC prognosis.

Despite these limitations, this study provides one of the most comprehensive and methodologically transparent evaluations of lipid-derived atherogenic indices in CRC, integrating tumor stage, metabolic background, and survival outcomes within a unified analytical framework, thereby helping to define the clinical boundaries of these biomarkers in oncologic practice.

### 4.2. What This Study Adds

• This study provides one of the first systematic evaluations of lipid-derived atherogenic indices in CRC while explicitly stratifying patients by T2DM, demonstrating that diabetes-related metabolic heterogeneity modifies tumor-associated lipid patterns.
• Using a stage-based analytical framework, we show that triglyceride-driven lipid indices (AIP, RC, and TyG index) vary across tumor stages in CRC patients but lose discriminatory capacity in CRC coexisting with T2DM patients.
• Despite clear metabolic differences by tumor stage and diabetes status, none of the lipid-derived indices independently predicted DFS or OS, highlighting the distinction between metabolic relevance and short-term prognostic utility in CRC.
• Triglyceride-driven markers (AIP, TG/HDL-C, and TyG index) appear more sensitive to CRC-related metabolic alterations than cholesterol-based indices, which are more strongly influenced by background metabolic status and therapy.

## 5. Conclusions

In conclusion, CRC coexisting with T2DM patients exhibits a markedly more atherogenic and insulin-resistant metabolic phenotype compared with CRC patients. While tumor stage influences certain lipid-derived indices in CRC patients, diabetes-related metabolic heterogeneity predominates in CRC coexisting with T2DM patients. Baseline lipid-derived indices did not demonstrate short-term prognostic utility for DFS or OS, highlighting their context-dependent interpretation and the need for longitudinal, integrative metabolic–oncologic studies.

## Figures and Tables

**Table 1:** Formulas utilized in the evaluation of IR and the metabolic profile.

Index	Formula
Atherogenic index of plasma (AIP)	log10 (TG/HDL-C) [25]
Atherogenic coefficient (AC)	(Total Cholesterol − HDL–C)/HDL–C [26]
Remnant Cholesterol (RC)	Total Cholesterol − LDL-C − HDL-C [27]
Triglyceride-glucose (TyG)	ln([TG (mg/dL) × Glucose (mg/dL)]/2) [28]

**Table 2:** General characteristics of patients (CRC vs. CRC coexisting with T2DM).

Characteristics	CRC Group (*n* = 180)	CRC Coexisting with T2DM Group (*n* = 60)
Age (yrs) (Median [IQR])	69.0 [62.0–73.0]	66.0 [64.0–69.0]
Gender, Female/Male (*n*)	11/14	29/31
Smoking (*n*)	83	29
Alcohol (*n*)	77	20
Hypertension (*n*)	35	52
T2DM duration (years)	-	11 [8–15]
Tumor extension (pT) (*n*)		
T1	9	4
T2	11	7
T3	125	32
T4	35	17
Regional lymph node metastasis (pN) (*n*)		
N0	103	22
N1	49	22
N2	28	16
Distant metastasis (pM) (*n*)		
M0	137	53
M1	43	7
TNM Stage of the WHO Classification of Tumors 2019 (*n*)		
I	19	12
II	75	14
III	43	27
IV	43	7
Tumor Grade (G) of the WHO classification of Tumors 2019 (*n*)		
G1	34	10
G2	105	14
G3	41	36
Tumor_Side (Right/Left) (*n*)		
Right	58	17
Left	122	43
Treatment		
Metformin (*n*)	-	60
Insulin (*n*)	-	30
SGLT2 or GLP1 (*n*)	-	24
Chemo Adjuvant (*n*)	90	43
Radiotherapy (*n*)	85	27
Complicated tumors (*n*, %)		
Obstruction	25 (27.47%)	11 (12.09%)
Perforation	15 (16.48%)	8 (8.79%)
Hemorrhage	6 (6.59%)	2 (2.19%)

CRC: colorectal cancer; TNM: tumor-node-metastasis; WHO: World Health Organization; G1: Well differentiated (low grade); G2: Moderately differentiated (intermediate grade); G3: Poorly differentiated (high grade); SGLT2: sodium-glucose cotransporter-2; GLP1: Glucagon-like peptide-1.

**Table 3:** Metabolic and biochemical profiles of patients (CRC vs. CRC coexisting with T2DM).

Parameters	CRC Group (*n* = 180)	CRC Coexisting with T2DM Group (*n* = 60)	*p*-Value
BMI (kg/m^2^) (Mean ± SD)	28.18 ± 4.38	26.77 ± 2.49	0.002
WC (cm) (Median [IQR])	99.30 [90.00–106.80]	106.50 [93.00–115.00]	0.023
HbA1c (%) (Median [IQR])	5.69 [5.38–5.89]	9.16 [7.58–10.38]	<0.0001
Glucose (mg/dL) (Median [IQR])	86.50 [78.00–96.46]	109.50 [89.05–130.80]	<0.0001
TC (mg/dL) (Median [IQR])	219.50 [178.00–254.00]	177.00 [143.30–220.30]	<0.0001
LDL-C (mg/dL) (Median [IQR])	131.70 [67.07–164.30]	115.10 [88.35–137.80]	0.006
HDL-C (mg/dL) (Median [IQR])	52.00 [43.00–64.55]	45.50 [37.00–51.75]	0.0001
non-HDL-C (mg/dL) (Median [IQR])	170.00 [119.70–193.00]	130.00 [91.25–164.50]	<0.0001
TG (mg/dL) (Median [IQR])	122.00 [89.50–181.30]	154.00 [104.50–224.30]	0.007

BMI: body mass index; WC: waist circumference; TC: total cholesterol; TG: triglycerides; HbA1c: glycated hemoglobin A1c.

**Table 4:** Lipid-derived indices of patients (CRC vs. CRC coexisting with T2DM).

Variable	CRC Group (*n* = 180)	CRC Coexisting with T2DM Group (*n* = 60)	*p*-Value
AIP (mmol/L) (Mean ± SD)	0.37 ± 0.30	0.55 ± 0.31	0.0002
AC (mg/dL) (Median [IQR])	3.08 [2.27–4.09]	2.87 [1.76–3.84]	0.2726
RC (mg/dL) (Median [IQR])	23.50 [17.61–36.25]	15.90 [17.70–49.25]	0.0142
TG/HDL (Median [IQR])	2.41 [1.52–3.59]	3.43 [2.28–4.98]	0.0003
non-HDL–C (mg/dL) (Median [IQR])	170.0 [119.70–193.00]	130.00 [91.25–164.50]	<0.0001
TyG (Mean ± SD)	8.60 ± 0.57	9.18 ± 0.64	<0.0001

**Table 5:** Gender-Based Differences in Lipid-Derived Indices of Patients (CRC vs. CRC coexisting with T2DM).

Variable	CRC Group (*n* = 180)			CRC Coexisting with T2DM Group (*n* = 60)		
	Male	Female	*p*-Value	Male	Female	*p*-Value
AIP (mmol/L) (Mean ± SD)	0.38 ± 0.30	0.38 ± 0.31	0.826	0.55 ± 0.28	0.56 ± 0.37	0.935
AC (mg/dL) (Median [IQR])	3.12 [2.31–3.93]	2.93 [2.15–4.19]	0.648	3.10 [1.77–4.15]	2.67 [1.74–3.75]	0.694
RC (mg/dL) (Median [IQR])	23.850 [18.20–37.20]	23.20 [16.80–34.00]	0.586	8.50 [−22.15–64.60]	19.00 [−12.90–40.00]	0.917
TG/HDL (Median [IQR])	2.50 [1.52–3.57]	2.39 [1.51–3.73]	0.847	3.43 [2.44–4.71]	3.62 [1.79–8.50]	0.932
non-HDL-C (mg/dL) (Median [IQR])	170.0 [119.70–193.00]	168.00 [117.10–192.100]	0.481	130.00 [92.75–164.50]	119.50 [84.50–176.50]	0.867
TyG (Mean ± SD)	8.58 ± 0.56	8.61 ± 0.59	0.767	9.06 ± 0.58	9.180 ± 0.74	0.527

**Table 6:** Lipid-derived indices across TNM stages (I–IV) of patients (CRC vs. CRC coexisting with T2DM).

Index	CRC Group (*n* = 180)						
	Stage I (*n* = 19)	Stage II (*n* = 75)	Stage III (*n* = 43)	Stage IV (*n* = 43)	Factor	F (DFn,DFd)	* p * Value
AIP (mmol/L) (Mean ± SD)	0.22 ± 0.33	0.43 ± 0.29	0.45 ± 0.25	0.27 ± 0.31	Row factor	F(74,102) = 0.968	0.555
					Column factor	F(3,102) = 4.122	0.008
AC (mg/dL) (Median [IQR])	2.30 [1.55–2.76]	3.38 [2.68–4.36]	3.54 [2.53–4.27]	2.55 [1.93–3.56]	Row factor	F(74,102) = 0.776	0.875
					Column factor	F(3,102) = 2.491	0.064
RC (mg/dL) (Median [IQR])	20.60 [14.40–37.40]	27.20 [19.60–37.50]	25.40 [19.40–38.80]	19.40 [14.20–27.20]	Row factor	F(74,102) = 0.978	0.536
					Column factor	F(3,102) = 2.889	0.039
TG/HDL (Median [IQR])	1.54 [0.82–2.79]	2.68 [1.74–3.95]	2.71 [1.90–3.59]	1.69 [1.04–3.19]	Row factor	F(74,102) = 1.138	0.270
					Column factor	F(3,102) = 1.648	0.183
non-HDL-C (mg/dL) (Median [IQR])	140.0 [108.40–182.90]	175.00 [134.20–202.00]	170.0 [132.00–193.00]	1418.00 [102.00–187.00]	Row factor	F(74,102) = 0.822	0.812
					Column factor	F(3,102) = 1.078	0.362
TyG (Mean ± SD)	8.41 ± 0.58	8.63 ± 0.55	8.76 ± 0.49	8.44 ± 0.62	Row factor	F(74,102) = 1.038	0.426
					Column factor	F(3,102) = 2.712	0.049
**Index**	**CRC Coexisting with T2DM Group** **(** ***n*** ** = 60)**						
	**Stage I** **(** ***n*** ** = 12)**	**Stage II** **(** ***n*** ** = 14)**	**Stage III** **(** ***n*** ** = 27)**	**Stage IV** **(** ***n*** ** = 7)**	**Factor**	**F** **(DFn,DFd)**	***p*** **Value**
AIP (mmol/L) (Mean ± SD)	0.43 ± 0.29	0.50 ± 0.23	0.58 ± 0.38	0.40 ± 0.20	Row factor	F(26,30) = 1.695	0.082
					Column factor	F(3,30) = 1.100	0.364
AC (mg/dL) (Median [IQR])	3.38 [2.68–4.36]	2.16 [1.66–3.76]	2.50 [1.76–5.06]	1.80 [1.61–3.02]	Row factor	F(26,30) = 2.120	0.025
					Column factor	F(3,30) = 1.567	0.218
RC (mg/dL) (Median [IQR])	27.30 [4.30–78.95]	14.90 [−23.60–44.90]	13.00 [−13.80–37.20]	−0.40 [−49.00–20.60]	Row factor	F(26,30) = 1.502	0.141
					Column factor	F(3,30) = 1.375	0.269
TG/HDL (Median [IQR])	2.68 [1.74–3.95]	3.27 [2.25–4.63]	3.50 [1.82–8.79]	3.14 [1.36–3.41]	Row factor	F(26,30) = 1.209	0.306
					Column factor	F(3,30) = 1.554	0.221
non-HDL-C (mg/dL) (Median [IQR])	142.50 [115.50–164.30]	126.50 [83.25–159.30]	130.00 [88.00–182.00]	192.00 [71.00–133.00]	Row factor	F(26,30) = 1.401	0.186
					Column factor	F(3,30) = 1.560	0.219
TyG (Mean ± SD)	8.63 ± 0.55	9.07 ± 0.54	9.06 ± 0.73	9.00 ± 0.29	Row factor	F(26,30) = 0.811	0.704
					Column factor	F(3,30) = 0.242	0.866

Distribution of lipid-derived indices across TNM stages (I–IV) in CRC patients without T2DM (CRC group) and with concomitant T2DM (CRC-T2DM group). A two-way ANOVA was used to assess the effects of the row factor (inter-individual variability) and the column factor (TNM stage) on each lipid-derived index. Significant column-factor effects indicate stage-dependent variation, whereas significant row-factor effects reflect heterogeneity unrelated to tumor stage. In the CRC group, TNM stage significantly influenced atherogenic index of plasma (AIP), remnant cholesterol (RC), and the triglyceride–glucose (TyG) index. At the same time, no significant stage-related effects were observed in the CRC-T2DM group. Data are presented as mean ± SD or Median [IQR], as appropriate.

**Table 7:** Kaplan–Meier estimates of overall survival (OS) according to T2DM status over follow-up time.

Time (Months)	No-T2DM Survival	T2DM Survival
12	0.983	1.000
24	0.899	0.950
36	0.857	0.914
48	0.838	0.914
60	0.838	0.914

Survival probabilities were estimated using the Kaplan–Meier method and are presented at predefined follow-up time points (12, 24, 36, 48, and 60 months) for patients without type 2 diabetes mellitus (T2DM) and those with T2DM. Differences between survival curves were assessed using the log-rank test (see Figure 1).

**Table 8:** Disease-free survival (DFS) according to lipid-related indices tertiles, stratified by T2DM status.

Subgroup	Biomarker	*n*	Events	Log-Rank χ^2^	Log-Rank df	Log-Rank *p*	T1 *n*	T1 Events	T2 *n*	T2 Events	T3 *n*	T3 Events
NoT2DM	AIP	180	23	4.022	2	0.134	60	12	60	6	60	5
NoT2DM	AC	180	23	2.716	2	0.257	60	10	60	9	60	4
NoT2DM	RC	180	23	3.254	2	0.197	62	12	58	6	60	5
NoT2DM	non-HDL-C	180	23	1.968	2	0.374	60	10	60	5	60	8
NoT2DM	TyG	180	23	1.228	2	0.541	60	10	60	6	60	7
NoT2DM	TG/HDL-C	180	23	4.022	2	0.134	60	12	60	6	60	5
T2DM	AIP	60	5	2.977	2	0.226	20	3	20	2	20	0
T2DM	AC	60	5	3.182	2	0.204	20	3	20	2	20	0
T2DM	RC	60	5	0.490	2	0.783	20	2	20	2	20	1
T2DM	non-HDL-C	60	5	2.154	2	0.341	20	3	20	1	20	1
T2DM	TyG	60	5	2.914	2	0.233	20	2	20	3	20	0
T2DM	TG/HDL-C	60	5	2.977	2	0.226	20	3	20	2	20	0

DFS was defined as the time from CRC diagnosis to death from any cause or last follow-up. Patients were stratified according to T2DM status. Within each subgroup (NoT2DM and T2DM), lipid-related indices, atherogenic index of plasma (AIP), atherogenic coefficient (AC), remnant cholesterol (RC), non-high-density lipoprotein cholesterol (non-HDL-C), triglyceride–glucose index (TyG), and triglyceride-to-HDL cholesterol ratio (TG/HDL-C), were categorized into tertiles based on subgroup-specific distributions. Kaplan–Meier survival curves were compared across tertiles using the log-rank test (three-group comparison). The number of patients and events in each tertile is reported. χ^2^ values, degrees of freedom (df), and corresponding *p*-values are shown for each comparison.

**Table 9:** Multivariable Cox proportional hazards models for DFS according to lipid-derived indices (per 1 SD increase).

All Patients (*n* = 240; Events = 28)			
**Lipid Index (per 1 SD)**	**HR**	**95% CI**	***p*** **-Value**
Model A (AIP)	0.714	0.480–1.064	0.098
Model B (AC)	0.722	0.436–1.196	0.206
Model C (RC)	0.660	0.389–1.120	0.123
**Stage III–IV Patients Only (** ***n*** ** = 120; Events = 28)**			
Model A (AIP)	0.823	0.548–1.236	0.348
Model B (AC)	0.848	0.508–1.414	0.527
Model C (RC)	0.780	0.463–1.314	0.350

AIP: atherogenic index of plasma; AC: atherogenic coefficient; RC: remnant cholesterol; DFS: disease-free survival. Lipid-derived indices were entered separately, one per model, as standardized continuous variables (per 1 SD increase) to avoid multicollinearity. Hazard ratios (HRs) were adjusted for age, sex, adjuvant chemotherapy, radiotherapy, and T2DM status.

**Table 10:** Multivariable Cox models for DFS in all patients: lipid-derived indices (per 1 SD) and clinical covariates.

All Patients (*n* = 240; Events = 28)			
**Model S1A—AIP (per 1 SD)**			
**Variable**	**HR**	**95% CI**	***p*** **-Value**
AIP (per 1 SD)	0.71	0.48–1.06	0.098
Age (years)	1.00	0.95–1.05	0.991
Male sex	0.59	0.27–1.26	0.171
Adjuvant chemotherapy	40.86	6.57–254.08	<0.001
Radiotherapy	0.25	0.07–0.82	0.023
T2DM	0.21	0.06–0.73	0.014
**Model S1B—AC (per 1 SD)**			
**Variable**	**HR**	**95% CI**	***p*** **-Value**
AC (per 1 SD)	0.72	0.44–1.20	0.206
Age (years)	1.00	0.95–1.05	0.872
Male sex	0.57	0.27–1.23	0.153
Adjuvant chemotherapy	49.51	8.14–300.99	<0.001
Radiotherapy	0.22	0.07–0.72	0.013
**Model S1C—RC (per 1 SD)**			
**Variable**	**HR**	**95% CI**	***p*** **-Value**
RC (per 1 SD)	0.66	0.39–1.12	0.123
Age (years)	1.00	0.95–1.05	0.872
Male sex	0.57	0.27–1.23	0.153
Adjuvant chemotherapy	49.51	8.14–300.99	<0.001
Radiotherapy	0.22	0.07–0.72	0.013
T2DM	0.21	0.06–0.73	0.014

AIP: atherogenic index of plasma; AC: atherogenic coefficient; RC: remnant cholesterol; DFS: disease-free survival. Lipid-derived indices were entered separately, one per model, as standardized continuous variables (per 1 SD increase). Models were adjusted for age, sex, adjuvant chemotherapy, radiotherapy, and T2DM status.

**Table 11:** Multivariable Cox models for DFS in patients with stage III–IV CRC: lipid-derived indices (per 1 SD) and clinical covariates.

Stage III–IV Patients Only (*n* = 120; *events* = 28)			
**Model S1D—AIP (per 1 SD)**			
**Variable**	**HR**	**95% CI**	***p*** **-Value**
AIP (per 1 SD)	0.82	0.55–1.24	0.348
Age (years)	1.03	0.97–1.10	0.344
Male sex	0.74	0.34–1.61	0.440
Adjuvant chemotherapy	21.80	3.86–123.14	<0.001
Radiotherapy	0.45	0.15–1.31	0.143
T2DM	0.21	0.07–0.63	0.006
**Model S1E—AC (per 1 SD)**			
**Variable**	**HR**	**95% CI**	***p*** **-Value**
AC (per 1 SD)	0.85	0.51–1.41	0.527
Age (years)	1.03	0.97–1.10	0.344
Male sex	0.74	0.34–1.61	0.440
Adjuvant chemotherapy	21.80	3.86–123.14	<0.001
Radiotherapy	0.45	0.15–1.31	0.143
**Model S1F—RC (per 1 SD)**			
**Variable**	**HR**	**95% CI**	***p*** **-Value**
RC (per 1 SD)	0.78	0.46–1.31	0.350
Age (years)	1.03	0.97–1.10	0.344
Male sex	0.74	0.34–1.61	0.440
Adjuvant chemotherapy	21.80	3.86–123.14	<0.001
Radiotherapy	0.45	0.15–1.31	0.143
T2DM	0.21	0.07–0.63	0.006

AIP: atherogenic index of plasma; AC: atherogenic coefficient; RC: remnant cholesterol; DFS: disease-free survival. Lipid-derived indices were entered separately, one per model, as standardized continuous variables (per 1 SD increase). Models were adjusted for age, sex, adjuvant chemotherapy, radiotherapy, and T2DM status. Stage III–IV-restricted analyses were performed as sensitivity analyses because no events occurred in early-stage disease.

## Data Availability

The data used to support the findings of this study are available from the corresponding author upon reasonable request.
